# Alcoholic liver steatosis in mice is aggravated by low-protein diet and reversed by FXR agonist

**DOI:** 10.1186/1753-6561-6-S3-P2

**Published:** 2012-06-01

**Authors:** Francislaine Aparecida dos Reis Lívero, Aline Maria Stolf, Arturo Alejandro Dreifuss, Amanda Leite Bastos-Pereira, Raphaella Chicorski, Inês Rabitto, José Ederaldo Queiroz Telles, Luiza Helena Gremski, Raílson Henneberg, Ronald PJ Oude Elferink, Alexandra Acco

**Affiliations:** 1 Federal University of Paraná, Curitiba, Paraná, 81530-980, Brazil; 2 University of Amsterdam, Amsterdam, 1105 BK, The Netherlands

## Background

Hepatic steatosis refers to the accumulation of triglycerides in hepatocytes, and it can be attributed to excessive ethanol consumption. The liver is the main organ of ethanol biotransformation and therefore, it can suffer with oxidative stress generated by ethanol. Since the FXR agonist 6ECDCA regulates adipose cell function, the aim of this work is to evaluate the participation of oxidative stress in ethanol-induced liver lesions and test the effects of FXR agonist against alcoholic liver steatosis development. For thus, diets with different amount of protein were used.

## Material and methods

Swiss male mice (8-10 weeks) were separated in 2 groups (n=24), which received liquid diet containing 10% ethanol or water (control group) for 6 weeks, as well as a low-protein diet (6%) or norm-protein diet (23%). In the last 15 days of the diet, mice that received ethanol or water were separated again for oral treatment, performing 8 groups (n=6) in the total. From these groups, 4 received FXR agonist 6ECDCA (3 mg.kg^-1^) and 4 received 1% tween (vehicle). Following this treatment, animals were anesthetized for sample collections (hepatic tissues and blood), in order to perform: serum biochemistry assays [aspartate aminotransferase (AST), alanine aminotransferase (ALT), cholesterol and triglycerides], hepatic oxidative stress (catalase, superoxide dismutase, glutathione-S-transferase, reduced glutathione and lipid peroxidation), liver histology (hematoxylin-eosin, Sudam black and Nile blue staining) and gene expression of *Srebp1f*, *FAS*, *PPARα*, *CYP7a1*, *HMGCoA reductase*, *ApoB*, *Scd1*, *p53* and *Bax.*

## Results

Ethanol associated with low-protein diet (6%) induced hepatic oxidative stress, increased plasmatic ALT and AST, and induced hepatic lipid accumulation. Many of these parameters were reverted by administration of 6ECDCA, including significant reduction in hepatic steatosis and improvement of antioxidant enzymes. These effects are possibly mediated by regulation of the genes *Srebpf1* and *FAS*, since both had the expression reduced by the FXR agonist.

## Conclusion

Ethanol induced intense hepatic steatosis when used in combination with low-protein diet (6%). Diet with regular amount of protein (23%) seems to prevent the hepatic effects of alcohol. Evaluating the participation of oxidative stress and FXR in the pathogenesis of alcoholic fatty liver disease in mice we demonstrated that 6ECDCA reverses the accumulation of lipids in the liver and decreases the hepatic oxidative stress. Thus, we speculate a possible therapeutic action of FXR agonists in alcoholic liver disease aiming to prevent the progression of this disease to more severe stages such as fibrosis, cirrhosis and hepatocellular carcinoma.

